# Plant disease detection model for edge computing devices

**DOI:** 10.3389/fpls.2023.1308528

**Published:** 2023-12-08

**Authors:** Ameer Tamoor Khan, Signe Marie Jensen, Abdul Rehman Khan, Shuai Li

**Affiliations:** ^1^ Department of Plant and Environmental Science, University of Copenhagen, Copenhagen, Denmark; ^2^ Department of Computer and Information Sciences, Pakistan Institute of Engineering and Applied Sciences, Islamabad, Pakistan; ^3^ Deparment of Information Technology and Electrical Engineering, University of Oulu, Oulu, Finland

**Keywords:** PlantVillage, deep learning, classifier, edge computing, MobileNetV3

## Abstract

In this paper, we address the question of achieving high accuracy in deep learning models for agricultural applications through edge computing devices while considering the associated resource constraints. Traditional and state-of-the-art models have demonstrated good accuracy, but their practicality as end-user available solutions remains uncertain due to current resource limitations. One agricultural application for deep learning models is the detection and classification of plant diseases through image-based crop monitoring. We used the publicly available PlantVillage dataset containing images of healthy and diseased leaves for 14 crop species and 6 groups of diseases as example data. The MobileNetV3-small model succeeds in classifying the leaves with a test accuracy of around 99.50%. Post-training optimization using quantization reduced the number of model parameters from approximately 1.5 million to 0.93 million while maintaining the accuracy of 99.50%. The final model is in ONNX format, enabling deployment across various platforms, including mobile devices. These findings offer a cost-effective solution for deploying accurate deep-learning models in agricultural applications.

## 1 Introduction

Plant diseases can be a major concern for farmers due to the risk of substantial yield loss. While applying pesticides can prevent or limit the impact of most plant diseases, their use should be restricted due to environmental considerations. Early and efficient detection of plant diseases and their distribution in the field is crucial for effective treatment. The implementation of automatic plant disease detection systems is, therefore, essential for efficient crop monitoring. Deep Learning Convolutional Neural Networks (CNNs) and computer vision are two developing AI technologies that have recently been employed to identify plant leaf diseases automatically.

Already in 1980, Fukushima (1980) presented a visual cortex-inspired multilayer artificial neural network for image classification. The network showed that the initial layer detects simpler patterns with a narrow receptive field, while later levels combine patterns from earlier layers to identify more complex patterns with wider fields. In 2012, Krizhevsky et al. (2012) developed the AlexNet architecture, which helped them win the ImageNet Large Scale Visual Recognition Challenge. Several CNN (Convolutional Neural Network) designs have been introduced since then Krizhevsky et al. (2012); Fu et al. (2018); Yang et al. (2023); Dutta et al. (2016); Sarda et al. (2021). These models are called “deep learning” architectures due to their 5-200 layers. Early investigations employed manually created characteristics from leaf picture samples. Later, the trends shifted to DCNN (Deep Convolutional Neural Network) architectures capable of effectively classifying data and automatically extracting features. Plant disease picture classification has been used to test a variety of CNN architectures Amara et al. (2017); Sladojevic et al. (2016); Setiawan et al. (2021); Yang et al. (2023); Qiang et al. (2019); Swaminathan et al. (2021); Schuler et al. (2022).

Plant disease diagnosis through image analysis employs various machine learning techniques Ferentinos (2018). These methods identify and classify diseases affecting cucumbers, bananas Fujita et al. (2016), cassavas Amara et al. (2017), tomatoes Ramcharan et al. (2017), and wheat Fuentes et al. (2017). Ramcharan et al. (2017) tested five architectures—AlexNet, AlexNetOWTBn, GoogLeNet, Overfeat, and VGG on 58 classes of healthy and sick plants. AlexNet achieved 99.06% and VGG 99.48% test accuracy. Despite the large variation in trainable parameters, these designs had test accuracy above 99%. Maeda-Gutiérrez et al. (2020) tested five architectures for tomato illnesses. All architectures tested had accuracies above 99%. However, when tested on field pictures, Ramcharan et al. (2017) encountered shadowing and leaf misalignment. These factors greatly affected classification accuracy.

Amara et al. (2017) classified banana leaf diseases using 60×60 pixel pictures and a simple LeNet architecture. Grayscale images had 85.94%, and RGB images had 92.88% test accuracy. Chromatic information Mohanty et al. (2016) is essential in plant leaf disease classification. Mohanty et al. (2016) used AlexNet and GoogLeNet (Inception V1) designs to study plant leaf diseases and found RGB images to be more accurate than their grayscale counterparts. Likewise, Schuler et al. (2022) split the Inception V3 architecture into two branches, one dealing with the grayscale part of the RGB image and the other branch dealing with the other two channels of the RGB image. The resultant architecture has 5 million trainable parameters and achieved an accuracy of 99.48% on the test dataset.

While these studies demonstrate the effectiveness of deep learning in plant disease classification, they often do not address the critical challenge of deploying these models on resource-constrained edge devices. In contrast, our work not only achieves high accuracy but also emphasizes optimizing deep learning models for such constraints. Recent advancements in the field substantiate this focus. For instance, Hao et al. (2023) discusses system techniques that enhance DL inference throughput on edge devices, a key consideration for real-time applications in agriculture. Similarly, the DeepEdgeSoc framework Al Koutayni et al. (2023) accelerates DL network design for energy-efficient FPGA implementations, aligning with our resource efficiency goal. Moreover, approaches like resource-frugal quantized CNNs Nalepa et al. (2020) and knowledge distillation methods Alabbasy et al. (2023) resonate with our efforts to compress model size while maintaining performance. These studies highlight the importance of balancing computational demands with resource limitations, a core aspect of our research. Thus, our work stands out by not only addressing the accuracy of plant disease detection but also ensuring the practical deployment of these models in real-world agricultural settings where resources are limited.

One major drawback in the broader field is that deep-learning approaches often have computational requirements, *i.e.*, higher memory and computing capacity, which are not always feasible for edge computing devices. Our paper tackles this challenge head-on, focusing on maximizing accuracy while operating within the resource constraints inherent to edge computing devices, thereby significantly enhancing the real-life applicability of deep learning models in agriculture.

The remaining part of the paper is organized as follows: Section 2 will look into the PlantVillage dataset, then we will explore the MobileNetV3-small architecture, model training, and finally, the post-training quantization. Section 3 will discuss the results and the comparison with existing methods. In Section 4, we will discuss the importance of the problem and the relevance of our results. Finally, Section 5 will conclude the paper with final remarks.

## 2 Materials and methods

### 2.1 PlantVillage dataset

**Figure 1:**
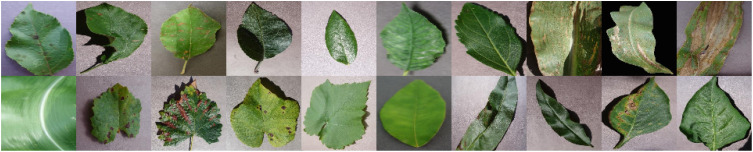
Sample images of the PlantVillage dataset. It is a diverse dataset with 14 plant species, including healthy and infected plants. The dataset includes a total of 54,309 image samples.

The present work used the publicly available PlantVillage-Dataset (2016). All images in the PlantVillage database were captured at experimental research facilities connected to American Land Grant Universities. The dataset included 54,309 images of 14 crop species, including tomato, apple, bell pepper, potato, raspberry, soybean, squash, strawberry, and grape. A few sample images of the plants are shown in **Figure 1**. It could be seen that some samples were healthy, and some were infected. There were 17 fungal infections, 4 bacterial diseases, 2 viral diseases, 1 mite disease, and 1 mold (oomycete). There were images of healthy leaves from 12 crop species, showing no obvious signs of disease. In total, the dataset included 38 classes of healthy and unhealthy crops. A detailed description of the distribution of species and diseases in the dataset is shown in **Table 1**. It included 14 crop species with 6 types, *i.e.*, fungi, bacteria, mold, virus, mite, and healthy. The dataset is imbalanced and not equally distributed across all 6 types.

**Table 1:** Distribution of observations in the PlantVillage dataset.

	Fungi	Bacteria	Mold	Virus	Mite	Healthy
Apple (**3172**)	1521					1645
Blueberry (**1502**)						1502
Bell Pepper (**2475**)		997				1478
Cherry (**1906**)	1052					854
Corn (**3852**)	2690					1162
Grape (**4063**)	3640					423
Orange (**5507**)		5507				
Peach (**2657**)		2291				360
Potato (**2152**)	1000		1000			152
Raspberry (**371**)						371
Soybean (**5090**)						5090
Squash (**1835**)	1835					
Strawberry (**1565**)	1109					456
Tomato (**18,162**)	5127	2127	1910	5730	1676	1592

The bold values represent the total number of images for that class in the dataset.

**Figure 2:**
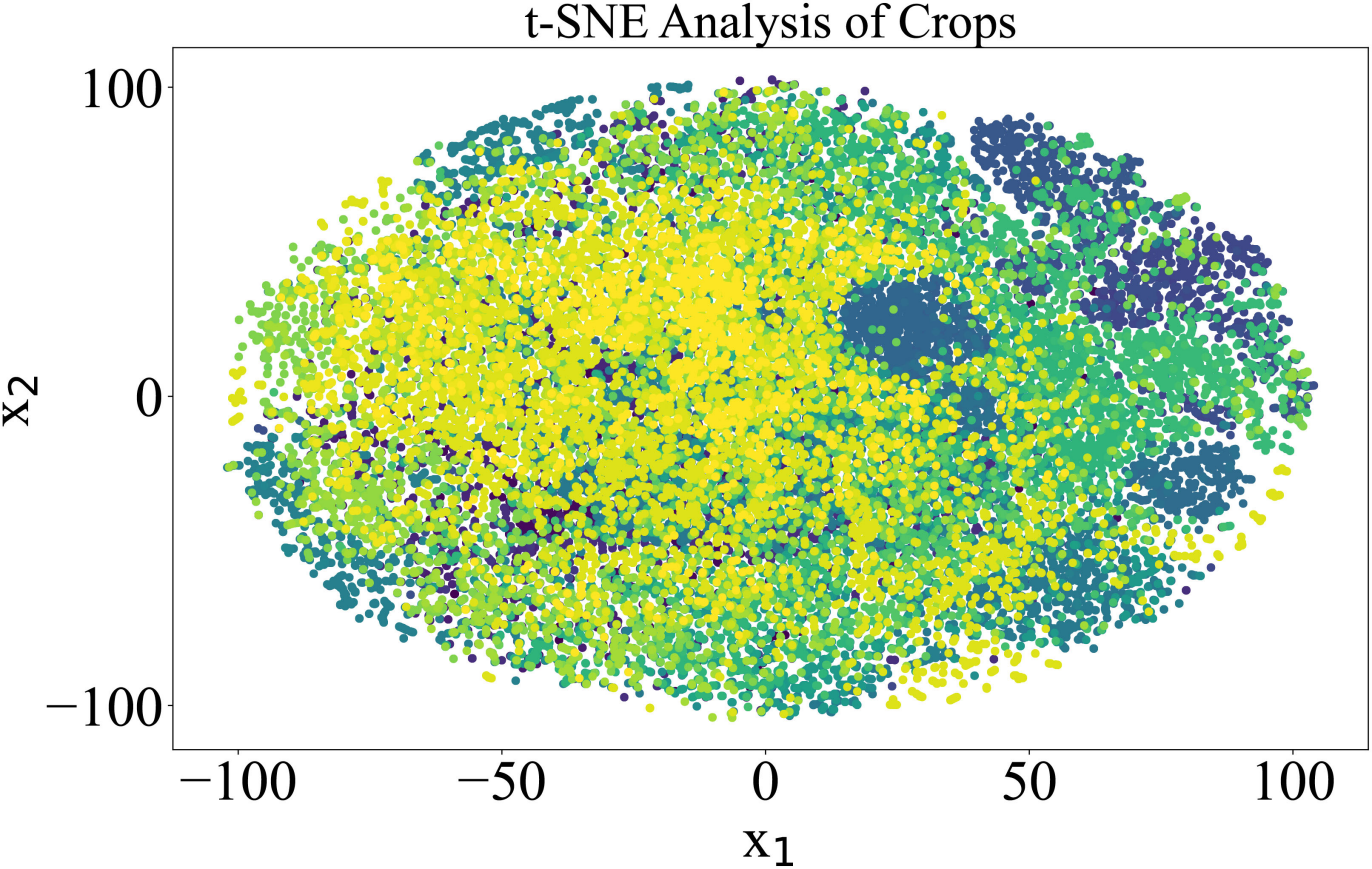
Visualization of the 38 classes in the PlantVillage data in two dimensions based on a t-SNE analysis. Each color in the spectrum represents one class in the PlantVillage dataset.

To further elaborate on the imbalanced nature of the dataset, t-SNE analysis was performed. t-SNE, or tDistributed Stochastic Neighbor Embedding, is a machine learning technique used to reduce dimensionality and visualize high-dimensional data. It attempts to represent complex, high-dimensional data in a lowerdimensional space while maintaining data point relationships. The data overlapping is quite visible in **Figure 2**, where the dimensions of the PlantVillage dataset were reduced to 2.

### 2.2 MobileNetV3-small

Recent research has focused on deep neural network topologies that balance accuracy and efficiency. Innovative handcrafted structures and algorithmic neural architecture search have advanced this discipline.

SqueezeNet used 1×1 convolutions with squeeze-and-expand modules to reduce parameters Iandola et al. (2016). Recent research has focused on minimizing MAdds (Million Additions) and latency instead of parameters. Depthwise separable convolutions boosted computational efficiency in MobileNetV1 Howard et al. (2017). MobileNetV2 added a resource-efficient block with inverted residuals and linear bottlenecks to improve efficiency Howard et al. (2018).

Later, MobileNetV3 Howard et al. (2019) extended MobileNetV2’s efficient neural network design. MobileNetV3’s backbone network, “MobileNetV3-Large,” used linear bottlenecks and inverted residual blocks to increase accuracy and efficiency. Hierarchical squeeze-and-excitation (HSqueeze-and-Excitation) blocks adaptively recalibrated feature responses in MobileNetV3. Hard-Swish and Mish activation functions balanced computing efficiency and non-linearity. MobileNetV3 used neural architecture search to find optimal network architectures.

MobileNetV3-small was created for resource-constrained situations. Its tiny, lightweight neural network system is efficient and accurate. MobileNetV3-small achieved this through architectural optimizations, a simplified design, and decreased complexity. A reduced network footprint reduced parameters and operations. MobileNetV3-compact solved several real-world problems with low computing resources or edge device deployment with a compact but efficient architecture. It introduced several key components to optimize performance and achieve high accuracy with fewer parameters.

#### 2.2.1 Initial convolution

An RGB image of size (*B,H,W*,3), where *B* is the batch size, *H* is the height, and *W* is the width, is used as an input. The image is passed through a standard convolutional layer with a small filter size (*e.g.*, 3x3) and a moderate number of channels (*e.g.*, 16).

#### 2.2.2 Bottleneck residual blocks

**Figure 3:**
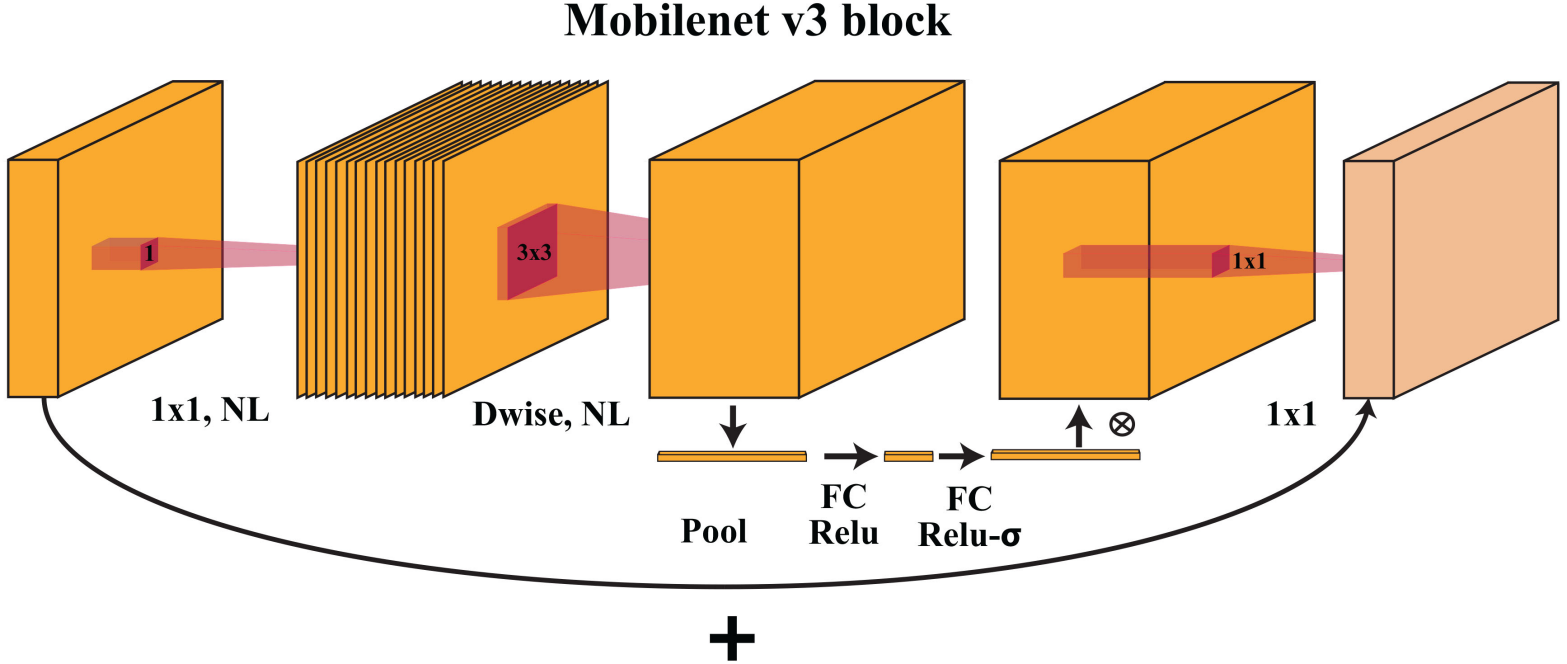
The MobileNetV3 block uses depthwise and pointwise convolutions to collect spatial patterns and integrate features. These blocks balance computing performance and precision, helping MobileNetV3 interpret complicated visual data.

MobileNetV3-small uses inverted bottleneck residual blocks, similar to its predecessor, MobileNetV2. The architecture is shown in **Figure 3**. Each block begins with a depth-wise convolution, which convolves each input channel separately with its small filter (*e.g.*, 3x3), significantly reducing the computational cost. The depth-wise convolution is followed by a point-wise convolution with 1×1 filters to increase the number of channels. A nonlinear activation function (*e.g.*, ReLU) is applied to introduce nonlinearity.

#### 2.2.3 Squeeze-and-excite module

The Squeeze-and-Excite (SE) module is incorporated into the MobileNetV3-small architecture to improve feature representation and adaptively recalibrate channel-wise information. The SE module contains two steps:

- Squeeze: Global average pooling is applied to the feature maps, reducing spatial dimensions to 1×1.
- Excite: Two fully connected (FC) layers are used to learn channel-wise attention weights. These weights are multiplied with the original feature maps to emphasize essential features and suppress less relevant ones.

#### 2.2.4 Stem blocks

MobileNetV3-small introduces stem blocks to further enhance feature extraction at the beginning of the network. The stem block consists of a combination of depth-wise and point-wise convolutions with nonlinear activation.

#### 2.2.5 Classification head

**Figure 4:**
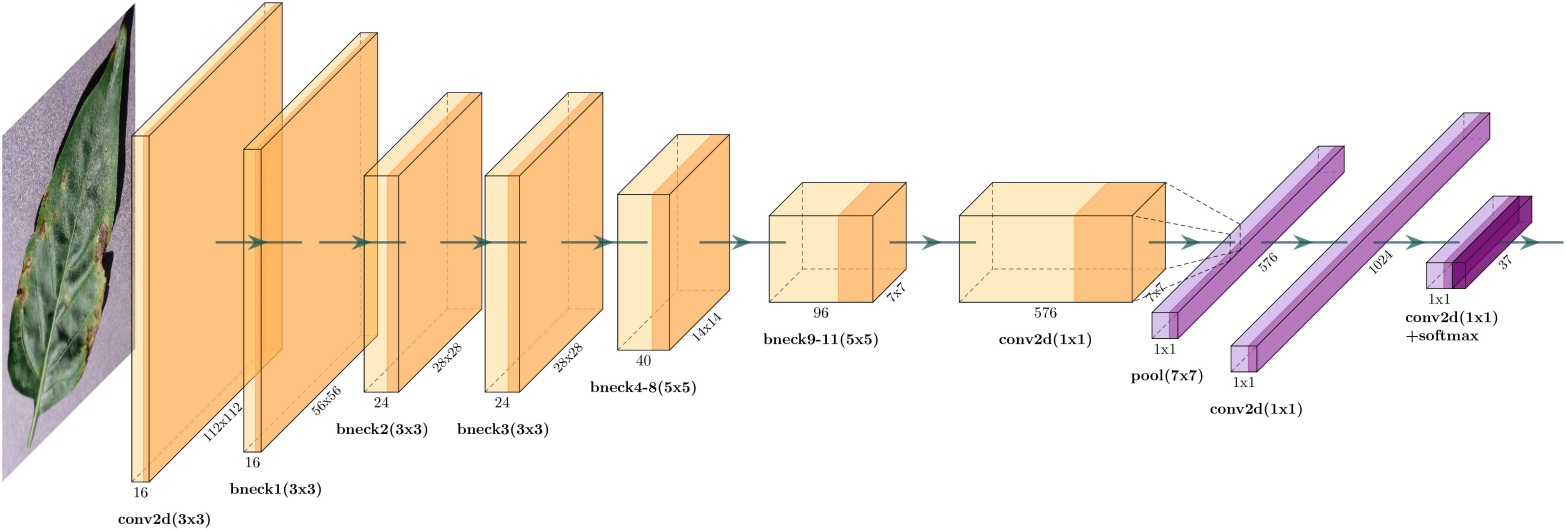
It shows the overall architecture of MobileNet-V3 Small. It includes a lightweight neural network design featuring depth-wise convolutions, inverted residuals, and a squeeze-and-excitation module for efficient feature extraction targeted for mobile and edge devices.

After multiple stacked bottleneck blocks and SE modules, the final feature maps are passed through a classification head to make predictions. Global average pooling is applied to the feature maps to reduce spatial dimensions to 1×1. The output of global average pooling is then fed into a fully connected layer with “softmax” activation to produce *K* class probabilities, as shown in **Figure 4**. The overall architecture is shown in **Table 2**.

**Table 2:** Specification of MobileNetV3-Small.

Input	Operator	Exp-Size	#Out	SE	NL	Stride
224 × 224 × 3	Conv2d, 3×3	-	16	-	HS*^b^*	2
112 × 112 × 16	BottleNeck, 3 × 3	16	16	✓	RE*^c^*	2
56 × 56 × 16	BottleNeck, 3 × 3	72	24	-	RE	2
28 × 28 × 24	BottleNeck, 3 × 3	88	24	-	RE	1
28 × 28 × 24	BottleNeck, 5 × 5	96	40	✓	HS	2
14 × 14 × 40	BottleNeck, 5 × 5	240	40	✓	HS	1
14 × 14 × 40	BottleNeck, 5 × 5	240	40	✓	HS	1
14 × 14 × 40	BottleNeck, 5 × 5	120	48	✓	HS	1
14 × 14 × 48	BottleNeck, 5 × 5	144	48	✓	HS	1
14 × 14 × 48	BottleNeck, 5 × 5	288	96	✓	HS	2
7 × 7 × 96	BottleNeck, 5 × 5	576	96	✓	HS	1
7 × 7 × 96	BottleNeck, 5 × 5	576	96	✓	HS	1
7 × 7 × 96	Conv2d, 1×1	-	576	✓	HS	1
7 × 7 × 576	Pool, 7 × 7	-	-	-	-	1
1 × 1 × 576	Conv2d, 1 × 1, NBN*^a^*	-	1024	-	HS	1
1 × 1 × 1024	Conv2d, 1×1, NBN	-	K	-	-	1

Conv 2 d, Convolution 2 DBottleNeck: Bottleneck Residual Blocks.

NBN, No Batch Normalization HS: Hard-Swish activation function.

RE, Rectified Exponential Linear Unit activation function Pool: Pooling Layer.

“✓” represents that squeeze-excitation (SE) layer is used in that bottleneck block and “-” represents SE-layer is not utilized.

The architecture focuses on reducing the number of parameters while maintaining competitive accuracy. The number of parameters in MobileNetV3-small is 1.5 million, which makes it suitable for deployment on resource-constrained devices and applications that require real-time inference.

### 2.3 Model optimization

Model optimization, or quantization, is an essential deep-learning technique that reduces a neural network’s memory footprint and computational complexity. Quantization enables efficient deployment on resource-constrained devices, such as mobile phones, peripheral devices, and microcontrollers, by converting the weights and activations of a full-precision model into lower-precision representations (e.g., 8-bit integers) Zhu et al. (2016). The procedure entails careful optimization to minimize the impact on model performance while achieving significant gains in model size reduction and faster inference times. Static quantization quantifies model weights and activations during training, whereas dynamic quantization quantifies model weights and activations based on the observed activation range at runtime.

For model quantization, the “Pytorch” built-in quantization tool was used Pytorch (2023). The PyTorch library’s torch.quantization.quantize dynamic function was used to dynamically quantify particular layers in a given classifier model. The torch.quantization.quantize dynamic function clones the input “model” before converting it into a quantized form. It then locates the cloned model’s layers corresponding to the requested classes, such as Linear (2D convolutional layers) and Conv2d (2D convolutional layers). The weights and activations of each recognized layer are subjected to dynamic quantization. The activations are quantized at runtime depending on the observed dynamic range during inference, whereas the weights are quantized to int8 (Integer stored with 8 bit). The cloned model replaces the quantized layers while leaving the other layers in their original floating-point format. Compared to the original full-precision model, the quantized model has less memory and better computational efficiency, and it is prepared for inference on hardware or platforms that support integer arithmetic.

While quantization is our chosen method, it is important to acknowledge that there are other effective techniques for compressing deep learning models. These include knowledge distillation, where a smaller model is trained to emulate a larger one Hinton et al. (2015), pruning, which involves removing less important neurons Han et al. (2015), and low-rank factorization, a technique for decomposing weight matrices Jaderberg et al. (2014). Each of these methods offers unique advantages in model compression and can be particularly beneficial in scenarios with limited computational resources. However, for the goals and constraints of our current study, quantization emerged as the most suitable approach.

The above technique was employed to quantize “Linear” and “Conv2d” layers with lower-precision representations, *i.e.*, 8-bit.

### 2.4 Model training

For the model training, the MobileNetV3-small model from PyTorch, trained on ImageNet data, was employed. The training pipeline was simple as it did not involve any preprocessing of the image data. The model was fed with PlantVillage images of resolution 224×224. The hardware specifications were as follows:

- Processor: 11th Gen Intel(R) Core(TM) i9-11950H @ 2.60 GHz 2.61 GHz
- RAM: 64 GB
- GPU: Intel(R) UHD Graphics & NVIDIA RTX A3000

Although the model was trained on a GPU, the final quantized model was intended for CPU and edge devices. The optimizer parameters were as follows:

- Optimizer: Adam optimizer
- Betas: (0.5,0.99)
- Learning rate: 0.0001

Some additional model-training hyperparameters included:

- Batch Size: 64
- Epochs: 200
- Training Data Percentage: 80%
- Validation & Test Data Percentage: 10% each.

## 3 Results

**Figure 5:**
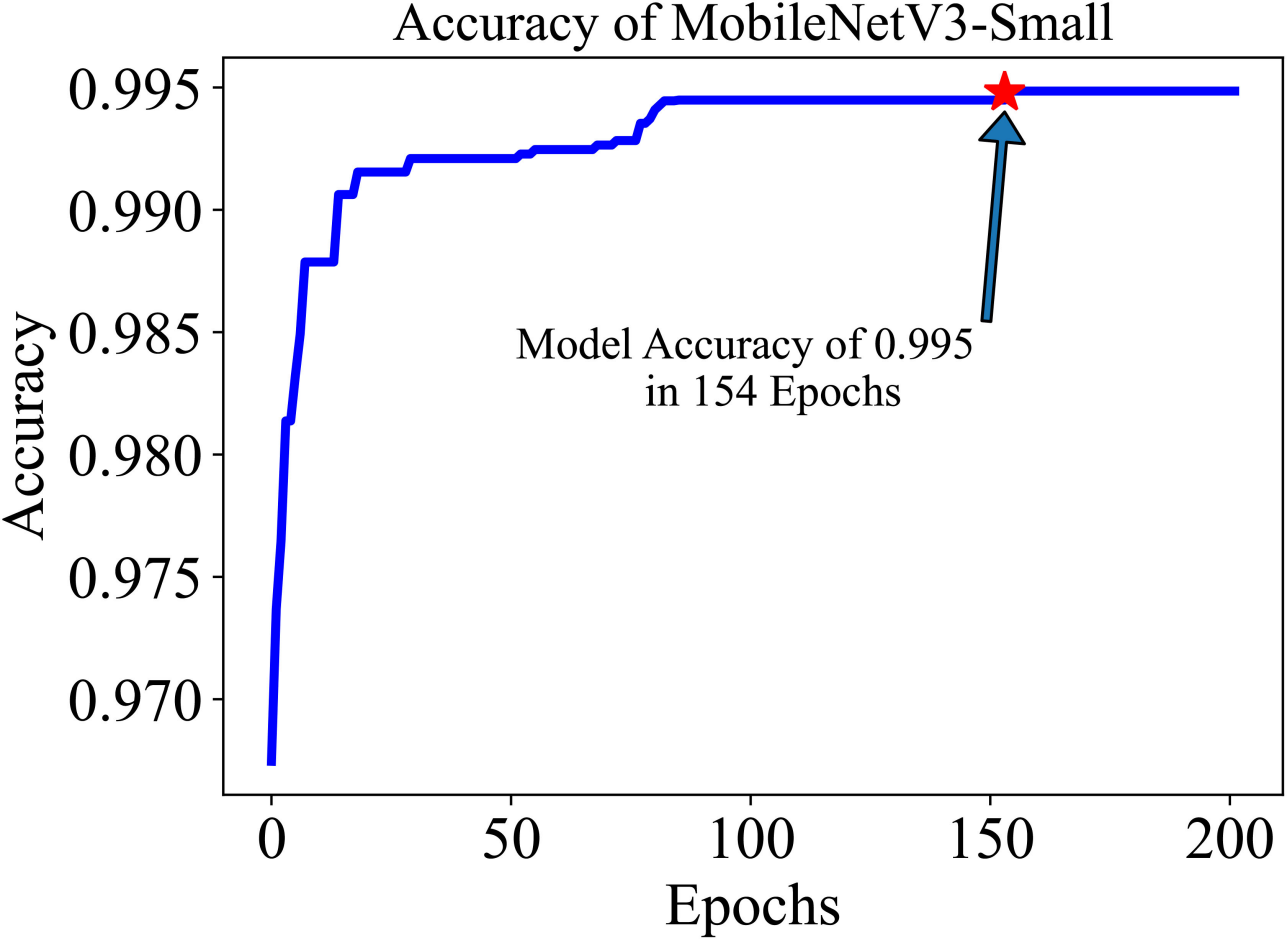
After training for 200 in epochs, the MobileNetV3-small gained an accuracy of 99.50 in roughly 154 epochs. The initial accuracy is approximately 97.0% because we used a pre-trained model.

**Figure 6:**
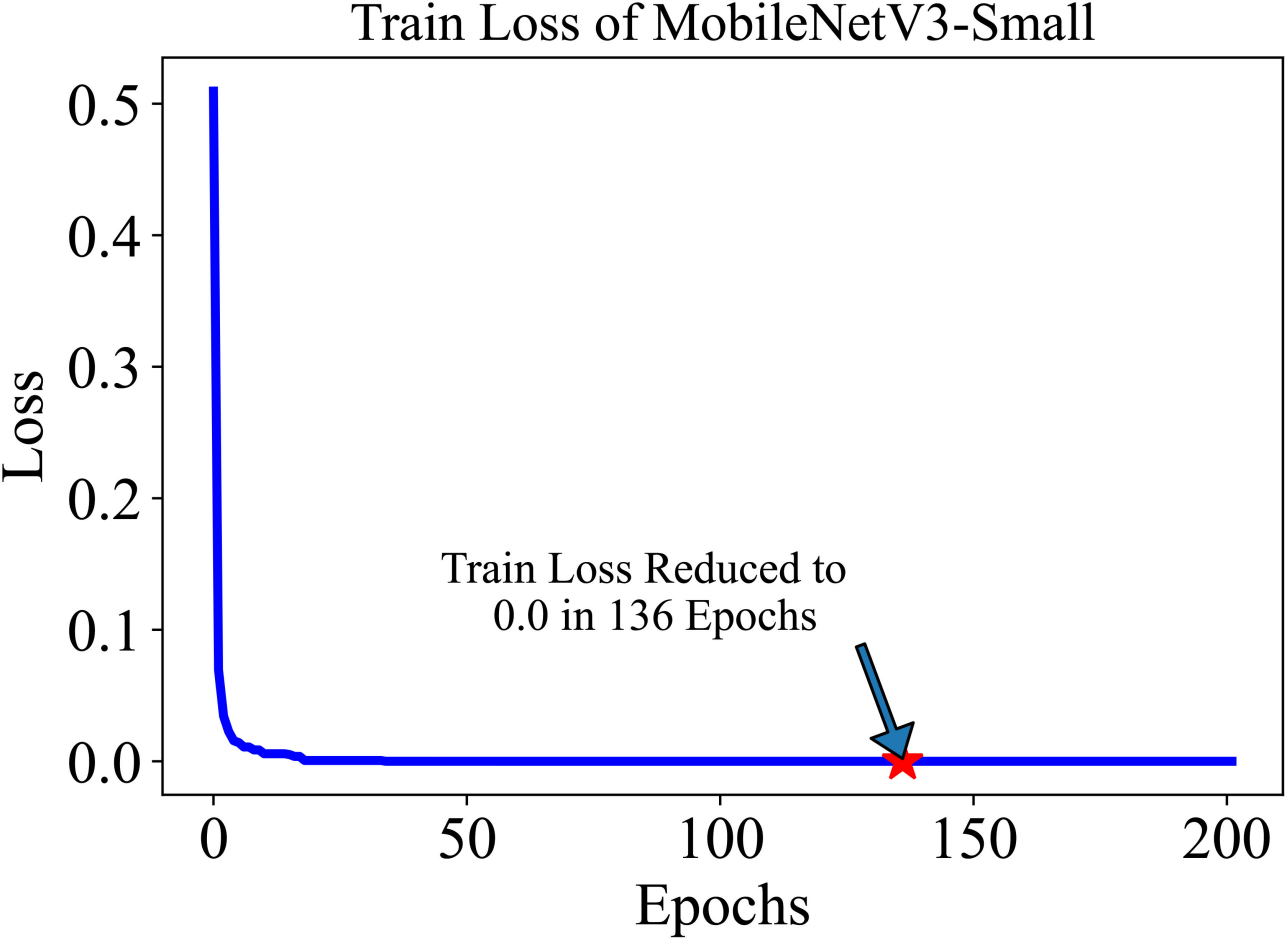
The training loss of MobileNetV3-small in 200 epochs quickly decreases and settles to 0.0 at 136 Epoch. The lower initial loss is the result of the pre-trained model.


The training and testing dataset included samples from all 38 classes. “Cross-entropy” was used as the loss function for the classification. The model’s performance was evaluated based on two key metrics: Accuracy (Equation 1) and F1 score (Equation 4). Accuracy, defined as the proportion of correctly identified classes to the total number of classes, reflects the overall effectiveness of the model in classification tasks. In our study, the initial accuracy of the pre-trained model was 97%, which increased to a maximum test accuracy of 99.50% at the 154-th epoch. This metric essentially gauges the model’s ability to label classes correctly. On the other hand, the F1 score, a harmonic mean of precision (Equation 2) (the proportion of true positive predictions in the total positive predictions) and recall (Equation 3) (the proportion of true positive predictions in the actual positive cases), measures the model’s ability to accurately identify positive examples while minimizing false positives. This metric is especially useful in understanding the model’s precision and robustness in identifying correct classifications without mistakenly labeling incorrect ones as correct. The trajectory of the model’s accuracy with MobileNetV3-Small is shown in **Figure 5**. Similarly, the training loss, *i.e.*, cross-entropy loss, rapidly approached 0 and was ultimately reduced to 0 at the 136-th epoch. The trajectory of the training loss for MobileNetV3-Small is depicted in **Figure 6**.

(Eq. 1) $$\text{Accuracy}=\frac{\text{Number of Correct Predictions}}{\text{Total Number of Predictions}}$$

(Eq. 2) $$\text{Precision}=\frac{\text{True Positives}}{\text{True Positives}+\text{False Positives}}$$

(Eq. 3) $$\text{Recall}=\frac{\text{True Positives}}{\text{True Positives}+\text{False Negatives}}$$

(Eq. 4) $$\text{F}1\text{ Score}=2\times \frac{\text{Precision}\times \text{Recall}}{\text{Precision}+\text{Recall}}$$

**Figure 7:**
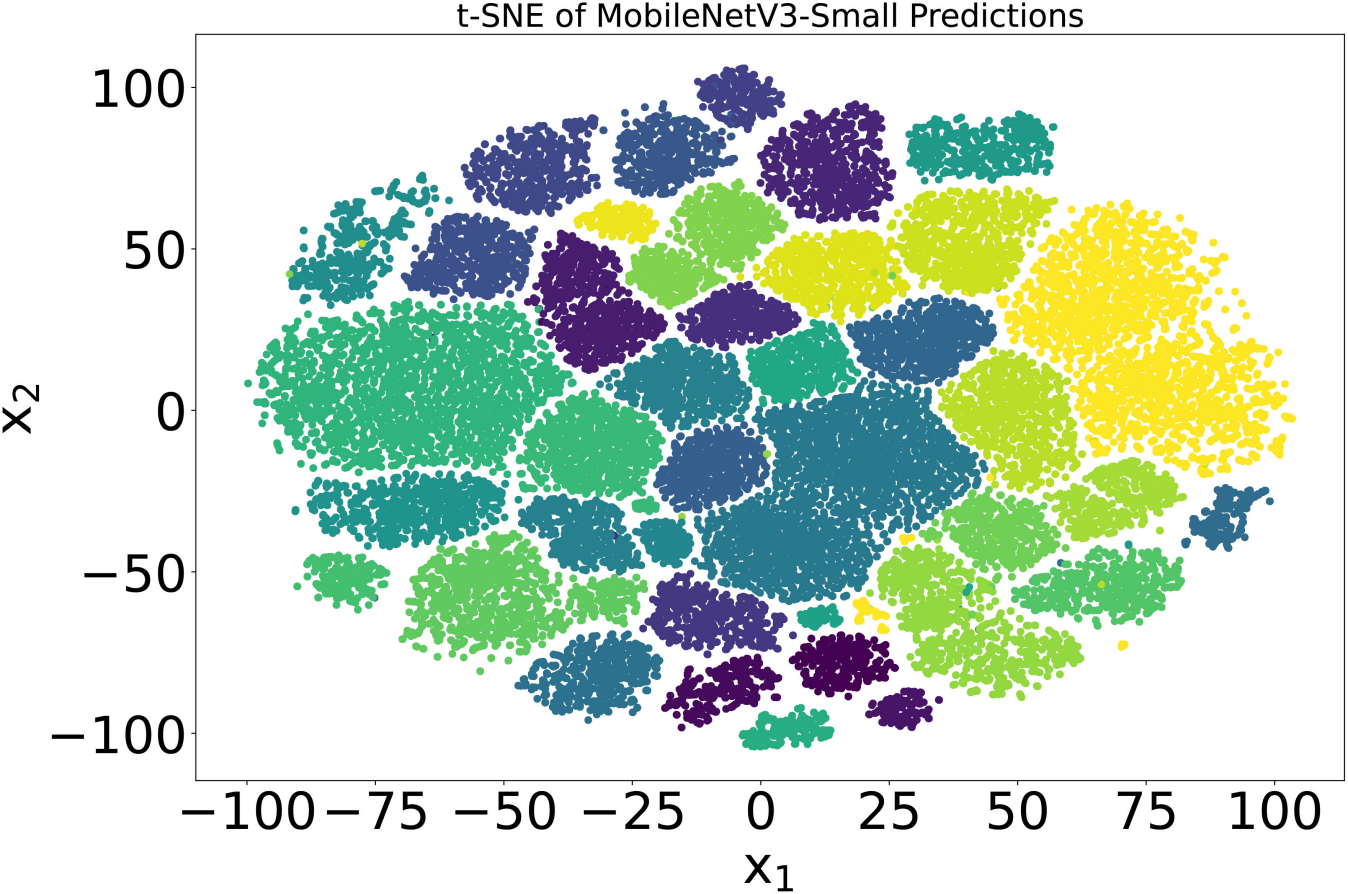
The t-SNE visualization of latent space of trained MobilenetV3-small model The output is from the second-last layer with a dimension of 1024, which is reduced to 2 using t-SNE. Each color in the spectrum represents one plant class in the PlantVillage dataset.

Later, the model was quantized, and the parameters were reduced to 0.9 million without reducing the accuracy of 99.50%. The inference time of the model was 0.01 seconds, and it achieved a frame rate of 100 frames per second (FPS) when running on a CPU. The higher-dimensional latent space of the model was also visualized using t-SNE Van der Maaten and Hinton (2008). 54,309 images of 38 classes were input to the trained model, and the output from the second-to-last layer of the MobileNetV3-small, which had dimensions of 1024, was obtained. Using t-SNE, the dimensions were reduced to 2, and the results were plotted to see the underlying classification modeling of the model. The results are shown in **Figure 7**. By forming distant clusters, it can be seen that the model efficiently classified 38 classes of plants.

Finally, the model was compared with other state-of-the-art architectures applied to the PlantVillage dataset. The comparison was based on three parameters, *i.e.*, the number of model parameters, model accuracy, and F1 score. The comparison is shown in **Table 3**. In the list of architectures, Schuler Schuler et al. (2022) had the highest accuracy and F1 score, and Geetharamani Geetharamani and Pandian (2019) had the least number of parameters, 0.2M. The proposed solution had the highest accuracy (99.50%) and F1 score (0.9950). However, the number of parameters was 0.9M, which was 5 times less than the model suggested by the Schuler et al. (2022) model.

**Table 3:** Results comparison on PlantVillage dataset.

Author	Architecture	Parameters	Accuracy	Fl-score
**Proposed**	**MobileNetV3-small**	0.9**M**	**99.50**%	**0.9950**
Schiller Schuler et al. (2022)	Inception V3 (Modifed)	5**M**	99.48%	0.9923
Mohanty Mohanty et al. (2016)	GoogLeNet	5**M**	98.37%	0.9836
Mohanty Mohanty et al. (2016)	AlexNet	60**M**	97.82%	0.9782
Toda Toda and Okura (2019)	Inception V3	5**M**	97.15%	0.9720
Geetharamani Geetharamani and Pandian (2019)	9 layers CNN	**0.2M**	96.46%	0.9815
Mohanty Mohanty et al. (2016)	GoogLeNet	5**M**	96.21%	0.9621
Mohanty Mohanty et al. (2016)	AlexNet	60**M**	94.52%	0.9449

TThe bold values correspond to the best value in each column.

## 4 Discussion

Large model sizes can pose significant challenges to their practical application in classification problems within agriculture. Such problems often necessitate real-time or near-real-time solutions, especially when identifying pests and diseases or assessing crop health. Bulky models can slow the processing of data, causing delays that might compromise timely interventions. Deploying these models on edge devices, frequently used in agriculture for on-site analysis, becomes problematic due to their computational and memory constraints. Furthermore, in regions with limited connectivity, transferring data for cloud-based processing by large models can be bandwidth-intensive, leading to additional lags. The energy and financial costs of running extensive models can also be prohibitive for many agricultural applications, especially for small-scale or resource-constrained farmers. Additionally, the adaptability of these models can be limited; training and fine-tuning them to cater to the diverse and evolving classification needs of different agricultural contexts can be challenging. In essence, while large models might boast superior accuracy, their size can often impede their practicality and responsiveness in addressing agricultural classification problems.

Previously proposed state-of-the-art solutions Schuler et al. (2022); Mohanty et al. (2016) for plant disease classifications achieve good accuracy. However, they have practical limitations in size and deployment. To overcome this issue, we proposed a solution with MobileNetV3-small. Its compact and efficient architecture enables rapid data processing, facilitating real-time agricultural interventions, such as pest detection or disease identification. The model’s low power consumption makes it ideal for battery-operated field devices, and its adaptability ensures relevance to diverse agricultural needs. Furthermore, its cost-effectiveness and ease of maintainability make it a practical choice for agricultural scenarios, offering a balance of high performance and resource efficiency.

While MobileNetV3 offers impressive efficiency and is optimized for edge devices, it has certain tradeoffs. The primary disadvantage is that, in pursuit of a lightweight and compact design, it might not always achieve the highest possible accuracy, especially when compared to larger, more complex models designed for high-performance tasks. This reduction in accuracy can be a limitation for applications where even a slight drop in precision can have significant consequences. Additionally, certain customizations or fine-tuning required for specific tasks might not be as straightforward, given its specialized architecture. Thus, while MobileNetV3 is advantageous for many scenarios, it may not be the best fit for situations demanding the utmost accuracy and complex model customizations.

The PlantVillage dataset, while comprehensive, exhibits an unbalanced nature with respect to the number of images available for different plant diseases. Unbalanced data can significantly impact deep learning model performance. Such datasets have extremely skewed class distributions, with one or a few classes having disproportionately more samples. This imbalance causes many issues. Deep learning models trained on unbalanced data tend to focus accuracy on the dominant class over the minority classes, biasing them towards the majority class. As a result, the model’s ability to generalize and forecast underrepresented classes falls, resulting in poor training and evaluation performance. Due to their rarity, the model may have trouble learning significant patterns from minority classes, making it less likely to recognize and classify cases from these classes.

MobileNetV3’s efficient and compact design offers a strategic advantage in addressing the imbalances inherent in datasets like PlantVillage. By leveraging transfer learning, a pre-trained MobileNetV3 is later fine-tuned on PlantVillage classes, harnessing generalized features to counteract dataset disparities. Its lightweight nature facilitates rapid training, enabling extensive data augmentation to enhance underrepresented classes. Furthermore, MobileNetV3 can serve as a potent feature extractor, with the derived features being suitable for synthetic sample generation techniques like SMOTE or ADASYN to achieve class balance. The model’s cost-effectiveness allows for swift iterative experiments, incorporating regularization techniques to deter overfitting dominant classes. Overall, MobileNetV3 presents a versatile toolset for researchers to navigate and mitigate the challenges of unbalanced datasets.

Training MobileNetV3 on the PlantVillage dataset and applying it to new images introduces challenges related to generalization. Absent categories, like healthy orange and squash, might be misclassified into familiar classes the model has seen. Diseases not in the training data, such as brown spots on soybeans, could be wrongly identified as another visually similar ailment or even as a healthy state. The model might also grapple with new images that differ in lighting, resolution, or background, especially if not exposed to such variations during training. The inherent class imbalance in the PlantVillage dataset, if unaddressed, can further bias the model towards overrepresented classes, affecting its performance on new or underrepresented classes. In essence, while MobileNetV3 is efficient, its accuracy on unfamiliar data hinges on the diversity and comprehensiveness of its training data.

Quantization compresses neural models by reducing the bit representation of weights and activations, enhancing memory efficiency and inference speed. “Weight quantization” reduces weight precision after training. This post-training quantization can introduce errors, as the model was not trained to accommodate the reduced precision. This can sometimes lead to a significant drop in model performance. Whereas “quantization-aware training” adjusts the model during training to a lower precision. PyTorch’s torch.quantization.quantize dynamic is notable, dynamically quantizing mainly the linear layers. This balances reduced model size and computational efficiency, preserving accuracy and making it apt for models with varied layer intensities.

The proposed pipeline, while efficient in its current application, does have certain limitations. Firstly, the pipeline is optimized for a specific dataset and task; scaling it to handle larger datasets or adapting it to different types of plants and diseases might require additional modifications. Secondly, the maintenance and updating of the model could present minor challenges. Ensuring that the model remains current with the latest data and continuously performs at its peak might necessitate regular updates and maintenance, which can be resource-intensive over time.

As we move forward from this study, we plan to extend our research to include a wider range of real-world datasets, such as those suggested by Tomaszewski Tomaszewski et al. (2023) and Ruszczak Ruszczak and Boguszewska-Mańkowska (2022). Our current focus on a controlled dataset lays the groundwork for this expansion. In future work, we aim to test and refine our models against the complexity of real-world agricultural scenarios, enhancing their generalization capabilities. This step-by-step approach, progressing from controlled conditions to more diverse datasets, aims to develop robust and adaptable deep-learning models for effective plant disease detection in practical agricultural settings.

## 5 Conclusion

The traditional and cutting-edge models have shown good accuracy; however, their suitability for onthe-ground applications with limited resources is often limited. By focusing on maximizing accuracy within resource constraints, we demonstrated the real-life usability of deep learning models in agricultural settings. Using the MobileNetV3-small model with approximately 1.5 million parameters, we achieved a test accuracy of around 99.50%, offering a cost-effective solution for accurate plant disease detection. Furthermore, post-training optimization, including quantization, reduced the model parameters to 0.9 million, enhancing inference efficiency. The final model in ONNX format enables seamless deployment across multiple platforms, including mobile devices. These contributions ensure that deep learning models can be practically and efficiently utilized in real-world agricultural applications, advancing precision farming practices and plant disease detection.

## Data availability statement

The original contributions presented in the study are included in the article/supplementary material. Further inquiries can be directed to the corresponding author.

## Author contributions

ATK: Methodology, Software, Writing – original draft. SJ: Supervision, Writing – review & editing. ARK: Conceptualization, Methodology, Validation, Writing – original draft. SL: Formal analysis, Methodology, Validation, Writing – review & editing.
